# Paravascular pathways contribute to vasculitis and neuroinflammation after subarachnoid hemorrhage independently of glymphatic control

**DOI:** 10.1038/cddis.2016.63

**Published:** 2016-03-31

**Authors:** C Luo, X Yao, J Li, B He, Q Liu, H Ren, F Liang, M Li, H Lin, J Peng, T F Yuan, Z Pei, H Su

**Affiliations:** 1 State Key Laboratory of Quality Research in Chinese Medicine, Institute of Chinese Medical Sciences, University of Macau, Macao, China; 2 Department of Neurology, National Key Clinical Department and Key Discipline of Neurology, The First Affiliated Hospital Sun Yat-Sen University, Guangzhou, China; 3 Department of Neurosurgery, Tangdu Hospital, Fourth Military University, Xi'an, China; 4 Zhongshan School of Medicine, Sun Yat-Sen University, Guangzhou, China; 5 School of Psychology, Nanjing Normal University, Nanjing, China

## Abstract

Subarachnoid hemorrhage (SAH) is a devastating disease with high mortality. The mechanisms underlying its pathological complications have not been fully identified. Here, we investigate the potential involvement of the glymphatic system in the neuropathology of SAH. We demonstrate that blood components rapidly enter the paravascular space following SAH and penetrate into the perivascular parenchyma throughout the brain, causing disastrous events such as cerebral vasospasm, delayed cerebral ischemia, microcirculation dysfunction and widespread perivascular neuroinflammation. Clearance of the paravascular pathway with tissue-type plasminogen activator ameliorates the behavioral deficits and alleviates histological injury of SAH. Interestingly, AQP4^−/−^ mice showed no improvements in neurological deficits and neuroinflammation at day 7 after SAH compared with WT control mice. In conclusion, our study proves that the paravascular pathway dynamically mediates the pathological complications following acute SAH independently of glymphatic control.

Cerebral aneurysm rupture causes subarachnoid hemorrhage (SAH), which is associated with a high mortality due to its secondary complications, including hemorrhage, hydrocephalus and delayed cerebral ischemia (DCI).^1, 2, 3^ Therapeutic interventions against the secondary complications, especially DCI, are yet limited, as the pathological mechanism underlying that is not fully understood.^2, 3, 4, 5, 6, 7^ Current hypotheses of the development of the secondary complications mainly include cerebral vasospasm (CVS) and the microcirculation disturbance, as well as parenchymal arterial lesions, microthrombosis and neuroinflammation.^1, 2, 4, 7, 8, 9^

Previous studies have shown that the blockade of cerebral lymphatic drainage deteriorated the secondary cerebral ischemia after SAH, suggesting that the cerebral lymphatic drainage pathway could be involved in the pathological mechanism of SAH.^10, 11^ However, the central nervous system (CNS) was considered lack of a conventional lymphatic drainage system in the past. Recently, several studies have shown that the brain has in fact the proper lymphatic system, including sinus-associated lymphatic vessels and the glymphatic system (GS).^12, 13, 14, 15^ Sinus-associated lymphatic vessels express all of the molecular hallmarks of lymphatic endothelial cells, contain cerebrospinal fluid (CSF) and immune cells, and drain into the deep cervical lymph nodes.^12, 13^

There is a histologically defined space in the brain, the Virchow–Robin space, where the subarachnoid space meets the paravascular space (or perivascular space in somewhere, PVS).^16^ The GS is a specialized brain-wide anatomic structure locating at the PVS surrounding the brain vasculature, which is ensheathed with the astroglial endfeet and astroglial water channel aquaporin-4 (AQP4).^14, 15^ The GS facilitates the efficient lymphatic clearance of extracellular solutes and fluid in the brain through astroglial-mediated interstitial fluid bulk flow.^14^

Impairment of GS involves neurological conditions including traumatic brain injuries,^17^ ischemic stroke^18^ and aged brain.^19^ Interestingly, brain imaging study with magnetic resonance imaging reported weakened GS perfusion following acute stroke or SAH.^18, 20^ However, little is known about whether the GS is involved in the secondary complications of SAH. Here, we examined the potential involvement of GS in SAH-associated pathology progression with *in vivo* two-photon microscopy and CLARITY technique.^21, 22^ Our data showed that subarachnoid blood flowed into the brain parenchyma rapidly through the PVS, causing CVS, vasculitis, widespread microinfraction and neuroinflammation in the animal model of SAH and SAH patients. Prevention of CVS with Fasudil^23^ did not improve the neurological impairment nor alleviated the pathology, while the PVS clearance with tissue-type plasminogen activator (tPA) infusion improved the behavioral recovery and reduced neuroinflammation in the brain. Interestingly, AQP4^−/−^ mice showed no improvements in neurological deficits and neuroinflammation at day 7 after SAH compared with WT control mice. Our study therefore suggested that the paravacular pathway dynamically mediates the pathological complications following acute SAH independently of glymphatic control.

## Results

### Intracranial pressure and physiological parameters monitoring

**Figure 1:**
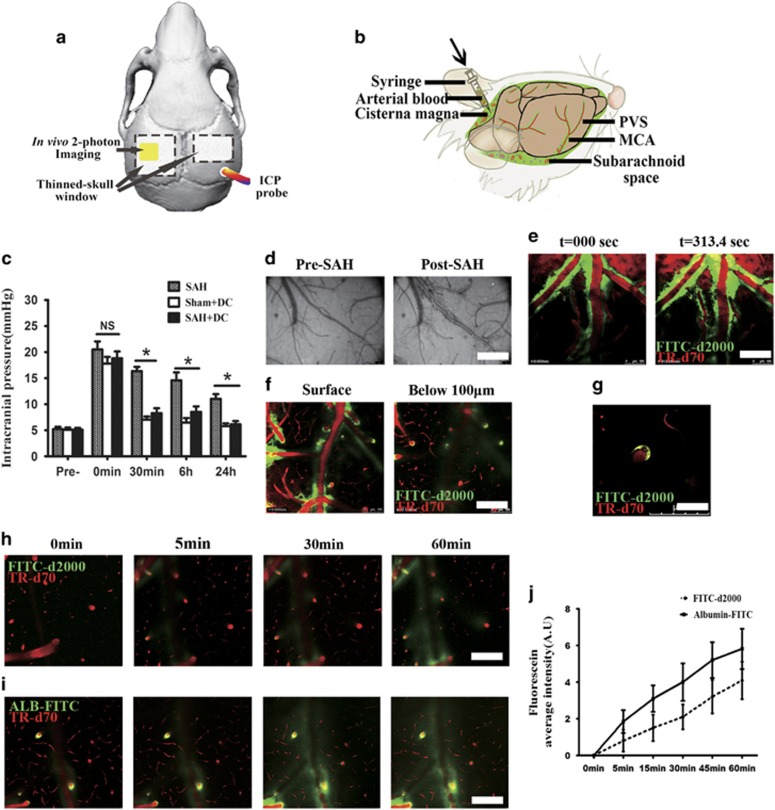
Subarachnoid blood flows into the brain parenchyma through the PVS after SAH in mice. (**a**) Schematic illustrating a decompresive thinned-skull window and ICP monitoring. (**b**) Schematic illustrating a SAH animal model established by injection of allogenic blood into cisterna magna. (**c**) Decompresive thinned-skull windows inhibited ICP elevation induced by intracisternal blood injection (*n*=5). (**d**) Representative images showing the appearance of the PVS surrounding the pia artery after SAH by a camera. (**e**) Representative images showing subarachnoid blood (labeled by FITC-d2000) flows into the PVS surrounding the pial artery (the bloodstream is defined by intravenously injected TR-d70) after SAH with two-photon imaging. (**f**) Representative images scanned at 100 *μ*m below the brain surface showing subarachnoid blood flows into the PVS surrounding penetrating arterioles (the bloodstream is defined by intravenously injected TR-d70). (**g**) Representative images showing the presence of a blood cell in the PVS of penetrating arterioles. (**h**) Representative images recorded the movement of FITC-d2000-labeled blood into the perivascular parenchyma along the penetrating arteriole over time. (**i**) Representative images recorded the movement of albumin (67 kDa) labeled with FITC (ALB-FITC) into the perivascular parenchyma along the penetrating arteriole over time. (**j**) The movement of ALB-FITC into the brain parenchyma was much faster compared with that of the FITC-d2000-labeled blood after the intracisternal injection (*n*=3). Scale bar: 60 *μ*m. Abbreviation: MCA, middle cerebral artery

SAH-induced elevated intracranial pressure (ICP) was considered closely related to early brain injury, decrease of cerebral blood flow (CBF) and global cerebral ischemia after SAH. To control the SAH-induced elevated ICP, we performed a decompressive (DC) thinned-skull window over both hemispheres before SAH as shown in Figure 1a. Different from the conventional thinned-skull window for microscopic observation (a 2 × 2 mm^2^ region over the target hemisphere), the decompressive thinned-skull window was generated over both hemispheres and much larger in area (4 × 4 mm^2^). The injection of blood or artificial CSF (aCSF) resulted in an immediate increase in ICP (from a baseline of 5.14 mm Hg to >25 mm Hg; *P*<0.001 *versus* Pre-SAH; Figure 1c). After 30 min and later on, ICP was significantly decreased in the animal with DC compare with the control (Figure 1c), indicating that the bilateral decompressive thinned-skull window effectively decreased the SAH-induced elevated ICP.

After two-photon microscopy *in vivo* imaging, physiological parameters (mean arterial blood pressure, blood gases, electrolytes and blood glucose) did not differ between SAH and sham-operated animals, indicating comparable physiological conditions in all investigated animals (Table 1).

### Subarachnoid blood flows into brain parenchyma via paravascular pathways after SAH

We firstly set up the SAH mouse model with infusing arterial blood (FITC-d2000 labeled) into cisterna magna (Figure 1b). We then performed live imaging with two-photon microscopy on the cortical middle cerebral artery region through a thinned-skull window. To investigate the blood flow at the initiation of SAH induction using two-photon microscopy, animals were immediately imaged after SAH induction rather than placed in a head-down position for 10 min. Within 5 min the blood components invaded the PVS along the pial artery (Figures 1d–g and Supplementary Video S1) and the penetrating artery below the brain surface (Figures 1f and g). The signal gradually increased in the following hour, and penetrated into the brain parenchyma, suggesting the fast diffusion of blood components via the paravascular pathway (Figure 1h). Similar phenomenon was observed in the SAH model using focused femtosecond laser pulses induced pial artery rupture (Supplementary Figure S1).

To confirm that natural serum proteins could also enter the parenchyma through the paravascular pathway, we labeled fresh arterial blood with albumin-FITC for injection into the cisterna magna. Intensive FITC signal was found in the parenchyma within 30 min after injection of albumin-FITC (Figure 1i) and its diffusion was much faster compared with that of the FITC-d2000 which has a bigger molecular weight (Figures 1h–j). All these results suggest that SAH leads to the paravascular pathway-mediated perfusion of blood components into the brain parenchyma.

### Blood components via the paravascular pathway induce neuroinflammation in the perivascular parenchyma after SAH

**Figure 2:**
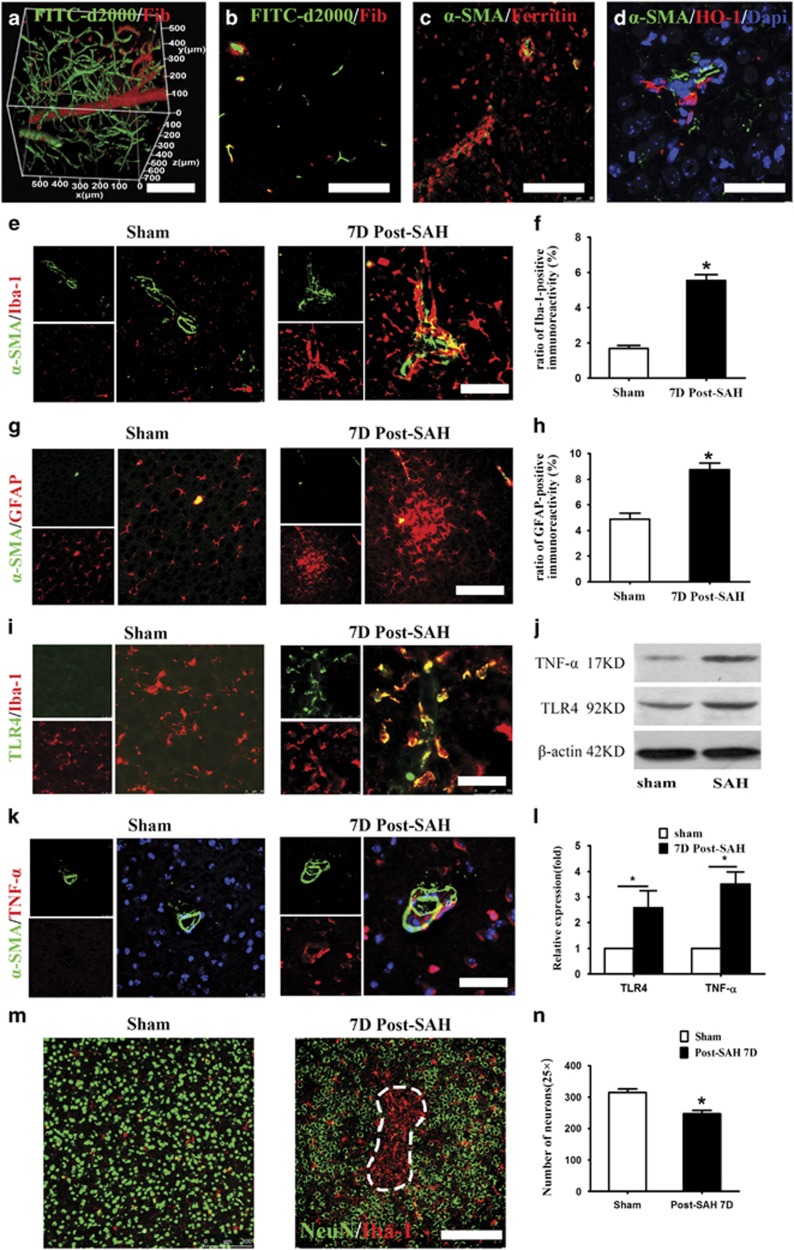
Influx of blood components via the paravascular pathway induces neuroinflammation and microcirculation dysfunction in the perivascular parenchyma after SAH. (**a**) Representative CLARITY images spatially showing the deposition of fibrinogen (red) in the extravascular zone along penetrating arterioles and their collaterals (labeled by intravenous injection of FITC-d2000) at 1 h after SAH. (**b**) A representative image under high magnifications showing the distribution of fibrinogen in the extravascular space surrounding the penetrating arteriole. (**c**) Immunostaining on brain sections showing the presence of ferritin in the perivascular parenchyma on day 7 after SAH. (**d**) Immunostaining on brain sections showing the expression of heme oxygenase-1 (HO-1) in the perivascular parenchyma on day 7 after SAH. (**e** and **f**) Microglial cells were pronouncedly activated in the perivascular parenchyma on day 7 after SAH (*n*=5, *P<0.05). (**g** and **h**) Astrocytes were pronouncedly activated in the perivascular parenchyma on day 7 after SAH (*n*=5, *P<0.05). (**i**) Representative images showing that activated microglial cells express TLR4 on day 7 after SAH. (**j**) Representative images showing higher expression of TNF-*α* in the perivascular parenchyma in SAH animals compared with the animals with sham surgery. (**k** and **l**) Western-blot analyses demonstrating significantly increased expression of TLR4 and TNF-*α* in the SAH animals compared with the animals with sham surgery (*n*=5, *P<0.05). (**m**) Representative images showing the cortical microinfarct with distinct infarction cores that were occupied by activated microglial cells and were devoid of neurons. (**n**) Quantitative analyses showed that the number of neurons in the cortex of SAH mice significantly decreased compared with the Sham *n*=5, *P<0.05). Scale bar: 200 *μ*m (**a**, **b**, **c**, **g** and **m**); and 75 *μ*m (**d**, **e**, **i** and **k**)

To histologically confirm the entrance of blood components into brain parenchyma through PVS, we applied the CLARITY technique to the mouse brain after SAH to reconstruct a 3D pattern of PVS-mediated diffusion and found that at 1 h after SAH, a large quantity of fibrinogen was deposited on the outer wall of penetrating vessels and their collaterals (Figure 2a). Immunostaining on brain slices demonstrated the presence of fibrinogen, ferritin and heme oxygenase-1 in the perivascular areas (Figures 2b–d), proving the permeabilization of blood components or their degradation products into parenchyma via the paravascular pathway.

We also observed robust responses of both microglial cells and astrocytes in the perivascular parenchyma of cortex and subcortex preferential adjacent to the ventricle at day 7 after SAH (Figures 2e–h and Supplementary Figure S2). Toll-like receptor 4 (TLR4) was highly expressed on the activated microglial cells after SAH (Figure 2i). A number of inflammatory factors including TNF-*α*, IL-1*β* and MCP-1 were remarkably increased in the perivascular parenchyma at day 7 after SAH (Figure 2k and Supplementary Figure S3). Western blotting analyses confirmed a significant increase in the expression level of TLR4 and TNF-*α* after SAH (Figures 2j and l). These results demonstrated extensive neuroinflammation in the perivascular parenchyma after SAH.

As early as 6 h after SAH, disrupted microcirculation was observed in the cortex based on two-photon imaging on dye-filling blood vessels (Figure 2m). The formation of microthrombi in the capillary network was detected at regions with disrupted blood circulation (Supplementary Figure S4 and Supplementary Video S2). At day 7 after SAH, histological examination revealed more neuronal loss in perivascular parenchyma of SAH animals compared with Sham animals (Figure 2n). Meanwhile, we identified microinfarction with distinct infarction cores that were occupied by activated microglial cells and were devoid of neurons in approximately one-third of SAH mice (total 6 mice) (Figure 2m).

### TPA but not Fasudil alleviates neurological deficits and neuroinflammation

**Figure 3:**
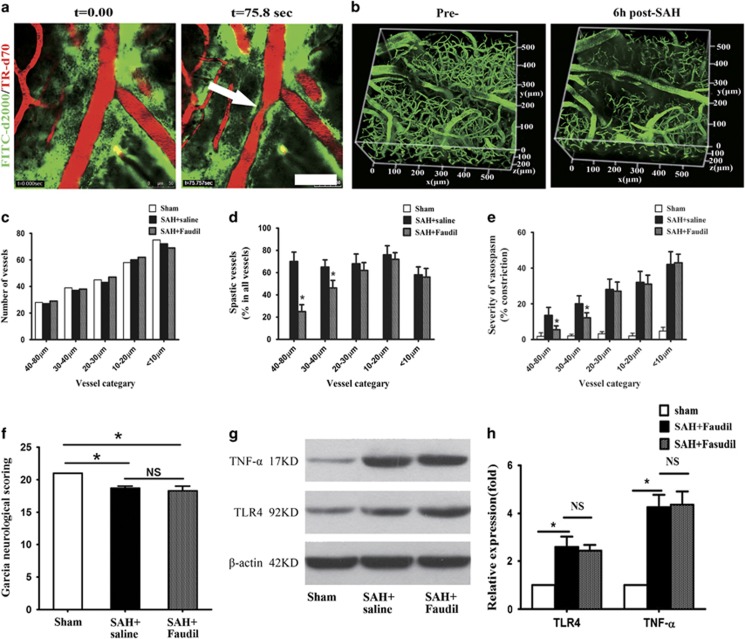
Prevention of vasospasm with Fasudil fails to alleviate neurologic deficits and neuroinflammation after SAH. (**a**) Representative images showing the vasospasm induced by the blood in the PVS. (**b**) Representative images showing the microvaspasm at 6 h after SAH. (**c**) The number of vessels was analyzed in each vessel category (5 mice in each experimental group). (**d**) Fasudil treatment was effective in reducing the incidence of large-artery (diameter of 40–80 *μ*m) vasospasm, but failed to the arterioles diameter less than 30 *μ*m. (**e**) Fasudil treatment was effective in reducing the severity of large-artery (diameter of 40–80 *μ*m) vasospasm, but failed to the arterioles diameter less than 30 *μ*m. (**f**) Fasudil treatment did not improve neurologic deficits on day 7 after SAH (*n*=5, *P<0.05; NS, *P*>0.05). (**g** and **h**) Western-blot analyses demonstrated that Fasudil treatment did not inhibit he expression of TLR4 and TNF-*α* after SAH (*n*=5, *P<0.05; NS, *P*>0.05). Scale bar: 50 *μ*m

Traditionally, CVS was believed to be a major cause of the secondary complications following SAH. Using the SAH animal model established by intracisternal injection of arterial blood (FITC-d2000 labeled), we confirmed that SAH could induce pial arterioles spasm (Figure 3a and Supplementary Video S3). A previous study has demonstrated that SAH could cause early and long-lasting microarterial constriction, especially obviously at 6 h after SAH.^24^ Similarly, numerous constricted microvessels were observed in a different arteriolar hierarchy at 6 h after SAH with two-photon *in vivo* imaging. Arterioles with a diameter of 40–80 *μ*m were prone to be affected by vasoconstriction, while small arterioles with a diameter less than 20 *μ*m were demonstrated to suffer from the severest constriction (Figures 3b–d). To evaluate the effectiveness of pharmaceutical reduction of vasospasm, we treated the SAH animals with Fasudil (a Rho-kinase inhibitor that is considered to have equally or even more effective than nimodipine in the prevention of CVS).^23, 25^ Fasudil administration was found to be effective in reducing the incidence and severity of arteriole vasospasm with large diameter (40–80 *μ*m), but showed no effects on the arterioles with the diameter less than 30 *μ*m (Figures 3b–d). Moreover, Fasudil administration failed to improve the neurological deficits and alleviate the neuroinflammation in the brain tissue at day 7 after SAH (Figures 3b–d). These results implied that the microvasospasm might be independent of large-artery vasospasm.

**Figure 4:**
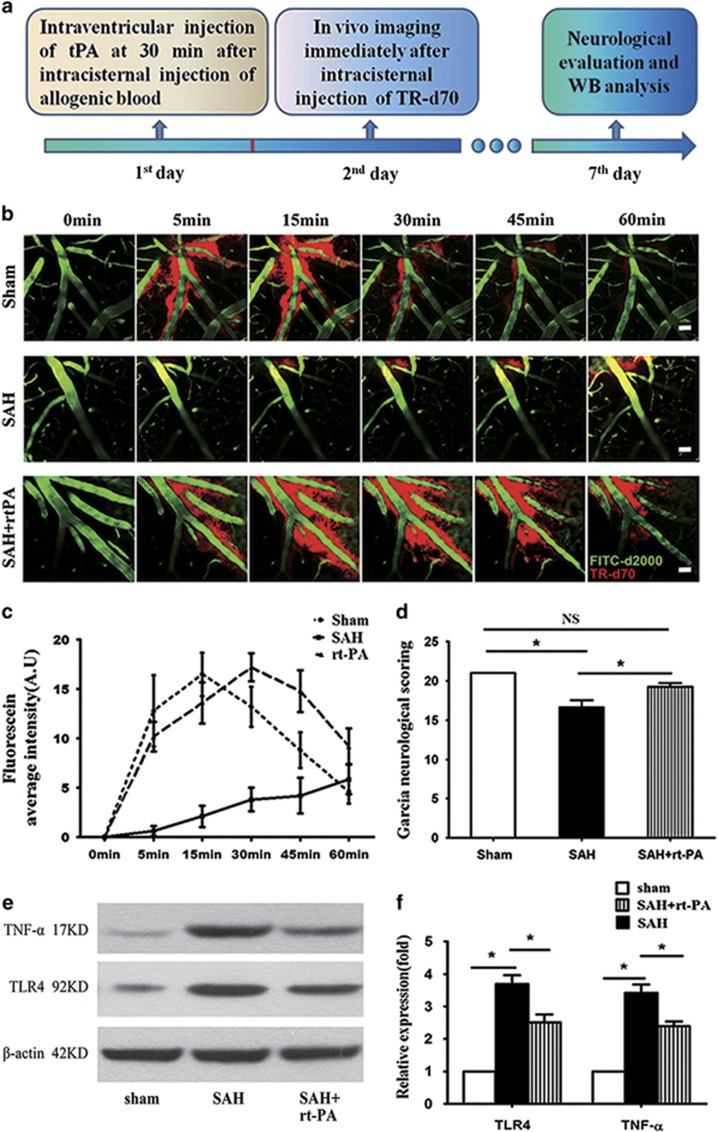
Administration of tPA alleviates neurological deficits and neuroinflammation after SAH. (**a**) Schematic illustrating the experimental protocol for tPA administration. (**b**) Blood cells in the PVS would aggregate to form clots and infusion of tPA is effective in GS clearance function. Upper row images showing intracisternally injected TR-d70 rapidly and efficiently entered the PVS over time in Sham animals; middle row images showing intracisternally injected TR-d70 was unable to enter the PVS in SAH animals; lower row images showing intracisternally injected TR-d70 could enter the PVS and be cleared away via the GS in SAH animals after tPA treatment; The bloodstream is defined by intravenously injected FITC-d2000. (**c**) Quantitative analyses show the movement of TR-d70 into the parenchyma via this GS was inhibited after SAH and tPA administration greatly improved the dye clearance function of the GS (*n*=3). (**d**) Quantitative analyses showed SAH animals treated with tPA significantly improved neurological deficits (*n*=5, *P<0.05; NS, *P*>0.05). (**e** and **f**) Western-blot analyses demonstrated SAH animals treated with tPA inhibited the expression of TLR4 and TNF-*α* (*n*=5, *P<0.05; NS, *P*>0.05). Scale bar: 25 *μ*m

The live imaging showed that blood cells aggregated to form blood clots within the paravascular space at 2–3 min after SAH (Figure 3a and Supplementary Video S3). We therefore wonder if targeting the clotted GS is beneficial to SAH animals (Figure 4a). We firstly found that after SAH, the dye-cleaning function of the paravascular pathway is severely impaired (Figure 4b), potentially due to the formation of blood clots within the paravascular pathway. We then infused tPA that is effective in blood clot clearance into the CSF, and found that the dye clearance function of the paravascular pathway was largely restored (Figures 4b and c). Excitingly, SAH animals treated with tPA showed significant improvement in neurological deficits and decrease in neuroinflammation on day 7 after SAH (Figures 4d–f), suggesting that the clotted paravascular pathway might contribute to the neuropathology of SAH *in vivo*.

### Reducing blood diffusion through the GS by deletion of AQP4 fails to ameliorate neurological deficits and neuroinflammation in SAH animals

**Figure 5:**
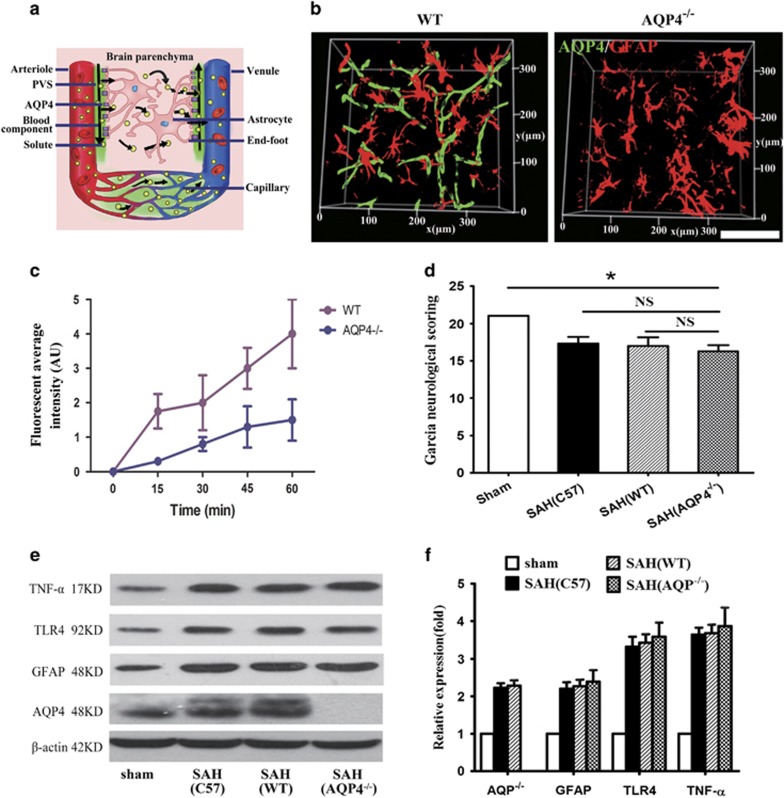
AQP4 knockout mice exhibit no improvement in neurologic deficits and neuroinflammation after SAH. (**a**) Schematic illustrating the role of AQP4 in the GS. In this brain-wide pathway, solute enters interstitial fluid (ISF) of the brain parenchyma along para-arterial route-dependent AQP4 water channel expressed on endfeet of astrocytes, then enters the bloodstream across the postcapillary vasculature or follows the walls of the draining veins to reach the cervical lymphatics. (**b**) Representative images of CLARITY showing the absence of AQP4 protein expression in AQP4^−/−^ mice. (**c**) The movement of intracisternally injected blood into the brain parenchyma was markedly reduced and retarded in Aqp4^−/−^ mice compared with WT control mice (*n*=5). (**d**) Garcia neurological scoring revealed that no improvement in neurologic deficits was found in AQP4^−/−^ mice compared with WT mice on day 7 after SAH (*n*=5, *P<0.05; NS, *P*>0.05). (**e** and **f**) Western-blot analyses demonstrated that no inhibition of the expression of GFAP, TLR4 and TNF-*α* was found in AQP4^−/−^ mice compared with WT mice on day 7 after SAH (*n*=5, *P<0.05; NS, *P*>0.05). Scale bar: 80 *μ*m

The AQP4 water channel expressed in perivascular astrocytic endfeet is a critical component in GS function (Figure 5a). Using AQP4 knockout mice, we investigated whether impaired GS could have an effect on neurological deficits after SAH (Figure 5b). We found that the infusion of intracisternally injected blood was reduced in the brain parenchyma in Aqp4^−/−^ mice, when compared with WT control mice (Figure 5c), suggesting that the impaired GS might prevent the blood components entry into the parenchyma. However, Aqp4^−/−^ mice showed no improvements in neurological deficits and neuroinflammation at day 7 after SAH compared with WT control mice (Figures 5d–f).

### Subarachnoid blood perfusion to the PVS in SAH patients

**Figure 6:**
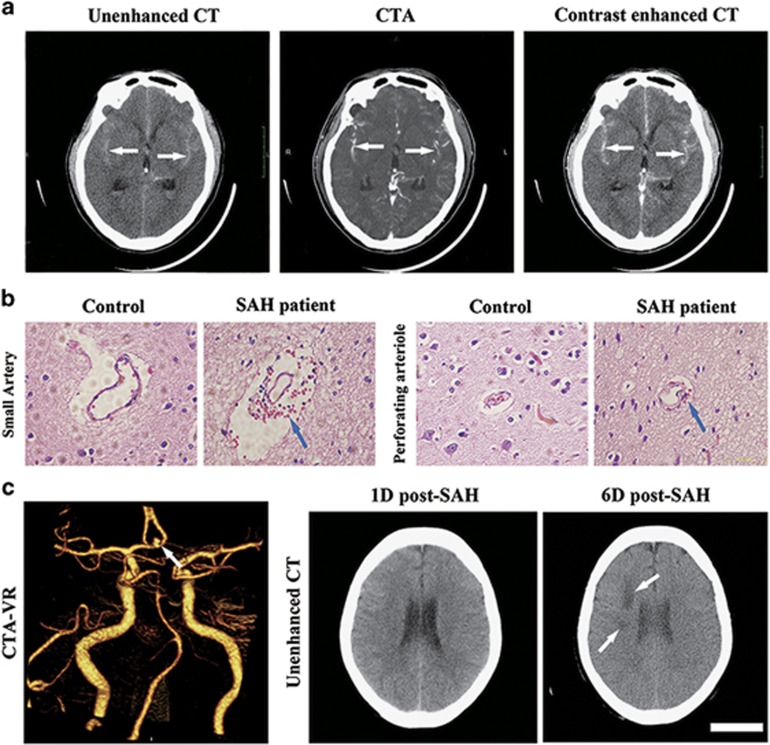
Subarachnoid blood perfusion into the GS in SAH patients. (**a**) Representative CT scanning images showing blood distribution in the GS in SAH patients. An unenhanced axial CT image (left) shows hemorrhagic densities in line shape in bilateral lateral fissures (arrows); a CTA axial image (middle) shows middle cerebral artery (MCA) in bilateral lateral fissures (arrows); an enhanced axial CT image (right) shows a highly coincidence between hemorrhagic densities and MCA in bilateral lateral fissures (arrows). (**b**) HE staining on patient brain sections showing the presence of unbroken blood cells in the GS after SAH (arrows). (**c**) Cerebral infarcts were found in SAH patients. CTA-VR shows the anterior communicating artery aneurysm in the patient (arrow); unenhanced CT scanning did not detect any obvious cerebral vasospasm at 3 h after SAH. However, two infarcts (33 mm × 14 mm and 14 mm × 9 mm in size, respectively; arrows) were detected in the right frontal lobe on day 6 after SAH. Scale bar: 6cm (**a** and **c**); 100 *μ*m (**b**)

We wonder if GS clots are detectable in human SAH patients as well. Therefore we performed retrospective analyses on computed tomography (CT) scan images of SAH patients who received the diagnosis and treatment in the First Affiliated Hospital of Sun Yat-Sen University in the past 2 years. Using unenhanced CT scan, CT angiography (CTA) and contrast-enhanced CT scan, we found the high-density areas were almost overlapped on these CT scan images in a set of SAH patients (Figure 6a), suggesting that the ferritin accumulation occurs along the blood vessels following SAH. We further obtained brain tissue samples from a SAH patient who received intracranial aneurysm clipping for hematoxylin–eosin (HE) staining and identified the deposition and aggregation of blood cells in the paravascular space of both the small artery and the perforating arteriole (Figure 6b). These results confirmed that in human SAH patients, blood perfusion through the PVS occurs as well.

When we retrospectively analyzed the CT scan images of SAH patients, we have identified some SAH patients with no early CVS (day 1 after SAH, unenhanced CT scan), however, showing infraction sites on day 6 (33 mm × 14 mm and 14 mm × 9 mm, respectively) (Figure 6c). These data suggested that blood perfusion through the PVS rather than early occurring CVS is contributing to the DCI and infraction occurrence.

## Discussion

SAH can cause focal or generalized brain dysfunction, including increased ICP, cerebral edema and swelling, blood–brain barrier damage, reduction in CBF, CVS, acute cerebral ischemia, acute and chronic hydrocephalus and DCI.^2, 26^ Among these devastating complications, DCI is an important cause for poor prognosis of SAH.^2^ It is generally believed that the toxic effects of blood in the subarachnoid space after SAH are the key factor leading to DCI.^27, 28, 29, 30, 31, 32^ In the present study, we found that, in SAH animal models, subarachnoid space blood quickly enters the paravascular pathway, with its components efficiently diffusing into the brain parenchyma. The rapid paravascular pathway of blood components entry into the brain parenchyma resulted in extensive perivascular neuroinflammation and microcirculation dysfunction throughout the brain. We also confirmed the distribution of blood components in the PVS in SAH patients by CT scan images and histological examinations. Thus, paravascular pathway plays a key role in mediating both the acute and delayed pathological complications following SAH.

It is commonly believed that CVS occurring at 4–10 days after SAH is the main reason for DCI, and that the reversal of CVS can improve prognosis of DCI.^2, 3, 7^ However, clinical trials targeting CVS over the past decades fail to show the desired effect.^4, 5, 7^ We found that Fasudil administration was effective in reducing the incidence and severity of large-artery (diameter of 40–80 *μ*m) vasospasm, but failed to the arterioles diameter less than 30 *μ*m, meanwhile failed to improve the neurological deficits and alleviate the neuroinflammation in SAH animals. In addition, it has been proposed that DCI probably results from microcirculation dysfunction after SAH.^2, 8, 9^ SAH-induced elevated ICP often caused intracranial circulatory decrease and was considered closely relation to microcirculation dysfunction.^8, 9^ In present study, we performed the bilateral decompressive thinned-skull window over both hemispheres to relatively control the SAH-induced elevated ICP. However, we still observed disrupted microcirculation and even the formation of microthrombi in the capillary network in the brain as early as 6 h after SAH, suggesting that a more complex mechanism of pathogenesis may be involved in microcirculation dysfunction.

Microcirculation dysfunction could result from perivascular neuroinflammation in the brain.^33^ In our SAH animal study we detected extensive perivascular neuroinflammation throughout the brain as revealed by pronounced activation of microglial cells and upregulation of TLR4, TNF-*α*, IL-1*β* and MCP-1 in the perivascular parenchyma. Consistently, other studies reported that there was an increased expression of TLR4 in both SAH patients and animals.^34, 35^ TLR4 is a pattern recognition receptor and can be activated by exogenous pathogenic microorganisms and foreign matters when passing through the blood–brain barrier.^36, 37, 38^ Its activation subsequently triggers the activation of nuclear factor-*κ*B and induces the expression of inflammatory factor.^36, 38, 39^ We therefore propose that blood components and their degradation products entering the perivascular parenchyma via the paravascular pathway activate TLR4 of microglial cells, which triggers the subsequent inflammation cascade after SAH.

AQP4 is the major water channel in the nervous system, and has been recognized as the critical functional component of the GS.^14, 40, 41^ Under normal circumstances, macromolecules are unable to pass through the AQP4 water channel expressed on the endfeet of astrocytes.^14^ In the present study, we found that FITC-d2000 that normally is confined in the PVS could enter the brain parenchyma after SAH, suggesting that SAH may change the PVS permeability and lead to more molecules to perfuse into the brain parenchyma. Using AQP4 knockout mice, we found that the genetic deletion of AQP4 significantly reduced and retarded blood moving into the brain parenchyma along the PVS. However, substantial decrease in blood diffusion from the PVS into the brain parenchyma did not alleviate neuroinflammation nor improve the neurological deficits after SAH. This indicates that the paravascular pathway may mediate vasculitis and neuroinflammation after subarachnoid hemorrhage independently of glymphatic control.

The recently discovered sinus-associated lymphatic vessels provided a conventional path for immune cells to exit the CNS, which performed the function of immune surveillance and might involve in diseases as diverse as the CNS infection and immune demyelination.^12, 13, 42^ Future work needs to further investigate whether sinus-associated lymphatic vessels are involved in vasculitis and neuroinflammation after SAH.

In the present study, we also found that the paravascular pathway would be blocked due to the formation of blood clots in the PVS after SAH. Administration of tPA after SAH significantly improved the GS clearance and alleviated neuroinflammation. In line with this, previous studies reported that application of tPA could significantly improve the GS dysfunction due to SAH^18^ and ameliorate cortical circulation.^20^ Furthermore, clinical studies confirmed that the intrathecal injection of thrombolytic agents can reduce the incidence of DCI and improve its prognosis.^40, 43^ Therefore, we propose that tPA application to CSF after SAH can improve the GS function and thus promote the clearance of unfavorable products in the brain tissue, thus significantly alleviating both the histological and the behavioral impairment after SAH.

**Figure 7:**
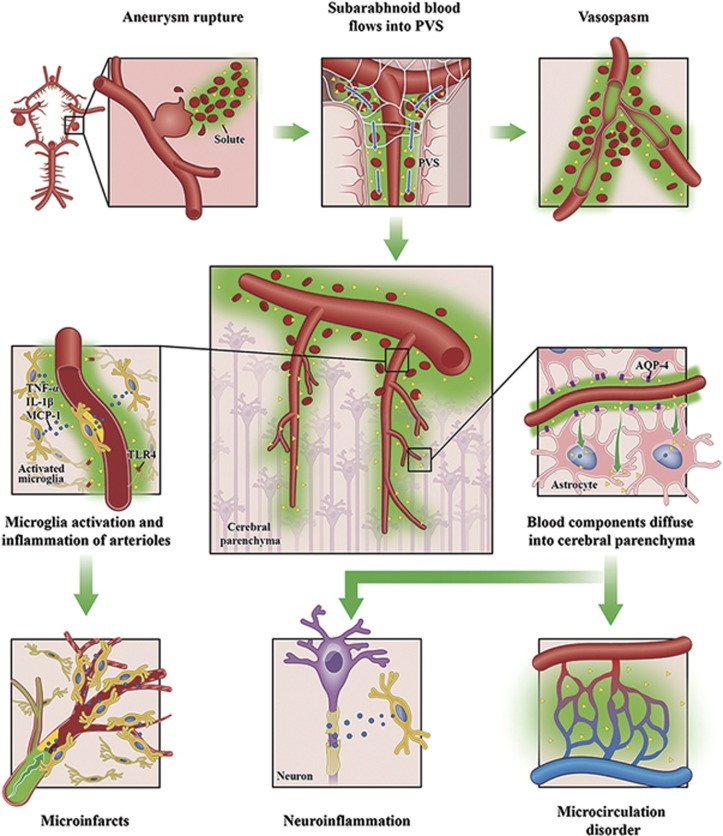
Schematic of the roles of the GS in mediating pathological complications following acute SAH. SAH is mostly caused by cerebral aneurysm rupture, leading to a large quantity of blood rushing into the subarachnoid space. Subarachnoid blood then rapidly enters the GS surrounding the cerebral vasculature, resulting into a cascade of devastating events: blood cells in the GS would aggregate or even form clots, which could induce CVS; its components and degradation products quickly diffuse into the perivascular parenchyma via the GS, leading to extensive perivascular glial activation, neuroinflammation, microcirculation dysfunction and even microinfarctions throughout the brain

In conclusion, our study confirms that the paravacular pathway plays important roles in mediating pathological complications following SAH, including CVS, DCI, parenchymal arterial inflammation, microcirculation dysfunction and widespread perivascular neuroinflammation independently of glymphatic control (Figure 7). The study provides a new perspective for understanding the pathological mechanism of SAH and suggests that the paravacular pathway targeted treatment might provide novel therapy against SAH.

## Materials and Methods

### Animals and monitoring

All animal protocols were approved by the ethical committee of the University of Macau. Unless otherwise noted, C57BL/6 mice weighing between 20 and 25 g and aging between 8 and 12 weeks were used in this study. We obtained Aqp4^−/−^ (Aqp4-null) breeders from Dr. Gang Hu (Jiangsu Key Laboratory of Neurodegeneration, Department of Pharmacology, Nanjing Medical University) and raised them in the Laboratory Animal Center, Sun Yat-Sen University. Mice were housed in a temperature-controlled, 12 : 12 light/dark room and were allowed free access to water and food.

Mice were deeply anesthetized with a combination of ketamine (0.12 mg/g intraperitoneally) and xylazine (0.01 mg/g intraperitoneally). To decrease the elevated ICP induced by SAH and image with *in vivo* two-photon microscopy, unless otherwise noted, the thinned-skull window over both hemispheres (left, 4 × 4 mm; right, 4 × 3 mm) was performed using a dental drill to a total skull thickness of approximately 20–30 *μ*m in all experimental animals before SAH induction (Figure 1a). ICP was measured in each animal at 0 min, 30 min 6 and 24 h after SAH. During the whole experiment, rectal temperature was maintained at 37±0.5 °C using a regulated heating pad with a rectal probe (TR-200; FST, CA, USA). A pulse oximeter clipped to the animal's hindpaw was used to monitor blood oxygen saturation and heart rate (MouseOx; Starr Life Sciences Corp., Oakmont, PA, USA). Blood gases and electrolytes were determined at the end of each experiment. Blood gas analysis was performed periodically and adjusted as needed to ensure physiological stability throughout the experiments. Subcutaneous injections of 5% glucose (wt/vol) in 0.3 ml saline were given every 2 h.

### Experimental groups

Sixty-five animals were assigned randomly to the following seven experimental groups: (1) Sham-operated group (*n*=13, 5 mice for immunofluorescence analysis, 3 mice for CLARITY analysis and 5 mice for western-blot analysis); (2) SAH induced by injection of fresh unheparinized arterial blood (*n*=13, 5 mice for immunofluorescence analysis, 3 mice for CLARITY analysis and 5 mice for western-blot analysis); (3) the PVS permeability group that received either TITC-d2000 (*n*=3) or albumin-FITC (*n*=3); (4) the tPA-treated group that was subdivided into control (SAH+aCSF, *n*=5) and tPA treatment (SAH+tPA, *n*=5); (5) the Fasudil group that was subdivided into control (SAH+saline, *n*=5) and Fasudil treatment (SAH+Fasudil, *n*=5); (6) the AQP4 group that was subdivided into WT (*n*=5) and AQP4^−/−^ (*n*=5); and (7) the SAH model induced by femtosecond laser injury (*n*=3).

### Animal models of SAH

SAH was induced by injection of fresh unheparinized arterial blood into the cisterna magna or intensively focused femtosecond laser pulses by a Ti:Sapphire laser (Chameleon Ultra II, Coherent Inc., CA, USA) to rupture the pial arteriole. For details, see Supplementary Materials and Methods.

### Two-photon imaging

Thinned skull window was prepared, and a Leica SP5 two-photon imaging system (Leica TCS SP5 MP CFS, Leica Microsystems, Mannheim, Germany) equipped with a Ti:Sapphire laser (Coherent Chameleon Ultra II), × 25/0.95 NA water-immersion objectives and Leica LAS X software was used to image the vasculature. Dynamic imaging was captured with 342 ms intervals using a XYT order for 1 h. Stacks of images were acquired using a step size of 1.0 *μ*m (single stacks) to a depth of 250 *μ*m in a XYZ order (512 × 512 pixels). For details, see Supplementary Materials and Methods.

### Clarity

Mice brain after perfused with formaldehyde acrylamide hydrogel were extracted for CLARITY processing. First, the brain was incubated in hydrogel monomer solution at 4 °C for 6 h and then in hydrogel monomer solution without 4% PFA at 4 °C for 3 days. The brain was then embedded in polymerized hydrogel at 37 °C for 3 h and cut into 2 mm-thick coronal sections with a mouse brain matrix. Clarification was completed by incubation in a solution of 8% (wt/vol) SDS (Sigma) in 0.1 M PBS (pH 7.5) at 37 °C for 2–3 weeks, followed by washing twice for 1 day in 0.1 M PBS+0.1% Triton X-100 (PBST; Sigma). For immunostaining and other details, see Supplementary Materials and Methods.

### Clinical data

We retrospectively analyzed 24 cases of hospitalized aneurysmal SAH patients who simultaneously underwent the examination of unenhanced cerebral CT scan, cerebral CTA and contrast-enhanced cerebral CT scan on admission from April 2013 to April 2015 in the First Affiliated Hospital of Sun Yat-Sen University. The diagnosis of SAH was based on the patient's medical history and clinical manifestation and verified by an examination of unenhanced cerebral CT scan. Among these 24 cases, there were 13 males and 11 females, with ages ranging from 19 to 68 years old. For details, see Supplementary Materials and Methods.

For human brain tissue sample data, the brain sample of SAH was isolated from a SAH patient (A 27-year-old female presented with a sudden severe headache was admitted to hospital on 6 April 2015. CT scan revealed a diffuse subarachnoid hemorrhage. CTA showed an aneurysm of the left former traffic artery) who received intracranial aneurysm clipping with right pterional approach on day 1 after SAH at Tangdu Hospital, Xi'An. The brain sample of control was isolated from a drug-resistant mesial epilepsy patient (a 21-year-old female presented with repeated psychomotor seizure for 3 years was admitted to hospital on 24 March 2015) who received temporal lobectomy on 27 March 2015 at Tangdu Hospital, Xi'An. This part of the study complied with the guidelines of the Declaration of Helsinki and was approved by the Human Ethics Committee of Tangdu Hospital, Xi'An, China. The written informed consent was obtained from the subject. The brain tissue sample was then proceeded for HE staining.

### Statistical analysis

All the data were presented as the mean±S.E.M. Statistical analysis was performed with SPSS 17.0 (SPSS, Inc., Chicago, IL, USA). Differences in perivascular Iba-1 and GFAP immunofluorescence were compared by an unpaired *t*-test. Differences in neurological scoring and western-blot analysis were evaluated by one-way ANOVA followed with Tukey's *post hoc* test for multiple comparisons. *P*<0.05 was considered to be statistically significant.

## Figures and Tables

**Table 1:** Mean arterial blood pressure (MAP), blood gases and electrolytes during two-photon microscopy

	**Sham**		**SAH**	
	**15 min**	**60 min**	**15 min**	**60 min**
MAP (mmHg)	69.5±8.25	65.8±9.78	67.8±8.2	65.8±9.24
PH (a.u)	—	7.26±0.08	—	7.24±0.06
pCO_2_ (mm Hg)	—	50.2±2.6	—	49.5±2.4
pO_2_ (mm Hg)	—	108.5±12.8	—	112.2±10.2
HCO_3_ (mmol/l)	—	21.2±2.8	—	20.8±1.8
Hb sat (%)	—	96.5±1.2	—	97.0±1.0
Na^+^ (mmol/l)	—	147.8±2.6	—	149.5±1.8
K+ (mmol/l)	—	5.0±0.6	—	4.9±0.8
Ca^2+^ (mmol/l)	—	1.38±0.16	—	1.42±0.12
Cl^−^ (mmol/l)	—	114.5±1.5	—	116.8±1.8
Glucose (mg/dl)	—	235.8±4.3	—	224.1±6.8

## Abbreviations

SAH: subarachnoid hemorrhage
tPA: tissue-type plasminogen activator
DCI: delayed cerebral ischemia
CVS: cerebral vasospasm
CNS: central nervous system
GS: glymphatic system
PVS: paravascular space
AQP4: aquaporin-4
CBF: cerebral blood flow
DC: decompressive
CSF: cerebrospinal fluid
aCSF: artificial cerebrospinal fluid
TLR4: Toll-like receptor 4
CT: computed tomography
CTA: CT angiography
