# Supplementary material for: Paravascular pathways contribute to vasculitis and neuroinflammation after subarachnoid hemorrhage independently of glymphatic control
Source: Cell Death Dis. 2016 Mar 31;7(3):e2160–. doi: 10.1038/cddis.2016.63 (PMC4823962; doi:10.1038/cddis.2016.63)
Supplement: Supplementary Information 1 [file cddis201663x1.doc]

**Supplemental Material**

**Materials and methods**

**Animals**

All animal protocols were approved by the ethical committee of the University of Macau. C57BL/6 mice weighing between 20 to 25g and aging between 8 to 12 weeks were used in this study. Aqp4^−/−^ (Aqp4-null) and WT mice were purchased from Nanjing Biomedical Research Institute, Nanjing University, China.

**Thinned-skull window preparation and Monitoring**

Mice were deeply anesthetized with a combination of ketamine (0.12 mg/g intraperitoneally) and xylazine (0.01 mg/g intraperitoneally). To decrease the elevated intracranial pressure (ICP) induced by SAH and image with *in vivo* 2-photon microscopy, unless otherwise noted, the thinned-skull window over both hemispheres (left, 4×4 mm; right, 4×3mm) was performed using a dental drill to a total skull thickness of approximately 20-30μm in all experimental animals before SAH induction (Fig. 1A). Special attention was paid to leave the remaining bone intact to serve as structural support for the thinned area.

For continuous monitoring of ICP, a cranial burr hole of the right hemisphere was made using a dental drill, and a microchip-based pressure transducer (Codman, Johnson & Johnson, UK) was introduced in the epidural space (Fig. 1A). ICP was measured in each animal at 0 minutes, 30minutes and 6h after SAH. The ICP probes were removed at the end of the monitoring period. During the whole experiment, rectal temperature was maintained at 37 ± 0.5°C using a regulated heating pad with a rectal probe (TR-200, FST, CA, USA). A pulse oximeter clipped to the animal’s hind paw was used to monitor blood oxygen saturation and heart rate (MouseOx; Starr Life Sciences Corp., Oakmont, PA, USA). Blood gases and electrolytes were determined at the end of each experiment. Blood gas analysis was performed periodically and adjusted as needed to ensure physiological stability throughout the experiments. Subcutaneous injections of 5% glucose (wt/vol) in 0.3 ml saline were given every 2h.

**Experimental groups**

Sixty-five animals were assigned randomly to the following 7 experimental groups: (1) Sham-operated group (n=13, 5 mice for immunofluorescence analysis, 3 mice for CLARITY analysis, and 5 mice for Western-blot analysis); (2) SAH induced by injection of fresh unheparinized arterial blood (n= 13, 5 mice for immunofluorescence analysis, 3 mice for CLARITY analysis, and 5 mice for Western-blot analysis); (3) PVS permeability group which received either TITC-d2000 (n=3) or albumin-FITC (n=3); (4) tPA-treated group which was subdivided into control (SAH + aCSF, n=5) and tPA-treatment (SAH + tPA, n=5); (5) Fasudil group which was subdivided into control (SAH + saline, n=5) and Fasudil-treatment (SAH + Fasudil, n=5); (6) AQP4 group which was subdivided into WT (n=5) and AQP4^-/-^ (n=5); and (7) SAH model induced by femtosecond laser injury (n=3).

**SAH models**

Unless otherwise noted, SAH model was induced by injection of fresh unheparinized arterial blood into the cisterna magna as described previously (1). In briefly, Mice were placed in a stereotactic frame. The skull of craniocervical junction and the atlanto-occipital membrane were exposed. A total of 60µL of arterial blood was withdrawn from the left ventricle of another anesthetized mouse using a 25 gauge needle and then injected into cisterna magna in 30 seconds. Animals were then immediately placed in a head-down position for 10 min to facilitate the diffusion of injected blood in the basal cisterns. Sham-operated animals underwent same procedures, except that aCSF (60μl), rather than blood, was injected into the cisterna magna. To evaluate the flow of blood after SAH, the blood was labeled with green fluorescence from the donor which was intravenously injected with 0.25ml Fluorescein isothiocyantate-dextran (FITC, 2000 kDa, FITC-d2000, 1.5% in saline, Sigma-Aldrich) or albumin labeled with FITC (albumin, 67KD, albumin-FITC, 5% in saline, Sigma-Aldrich) through the tail vein. Overall, the mortality in this study was 7.1% (totally 70 mice) and most often occurred immediately following SAH induction. Only mice who survived the initial surgery were included in this study. After SAH surgery, animals recovered while being observed for health and pain in a private cage where food and water were freely available.

For mimicking the aneurysmal SAH, we applied intensively focused femtosecond laser pulses by a Ti:Sapphire laser (Coherent Chameleon Ultra II, CA) tuned at 800nm with 140fs pulse width and 80 MHz reputation rate, leading to rupture of the pial arteriole. Briefly, the edge of the lumen of target arteriole was selected at the appropriate optical zoom setting for femtosecond laser ablation at the 2-photon wavelength of 800nm. Then, the wall of the arteriole was irradiated with 800nm laser, which intensity (Max. power 3.5W) was controlled by EOM setting at 60% (roughly 2.1W) for 30 seconds until rupture of artery wall was observed, which was indicated by a mass of extravasation of fluorescently labeled plasma outside the vessel. If no injury was observed, the energy was increased by ～10% and the process was repeated. Once artery rupture occurred, the dynamic imaging was captured with 342ms intervals using a XYT order for 1h.

***In vivo* 2-photon Imaging**

To evaluate the blood flow after SAH, the animal with a thinned-skull window was fixed with a custom-fabricated metal frame by holding the head with a cyanoacrylate and dental cement, and then fixed on the stage of Leica DM6000 CFS. To visualize the vasculature, 0.25ml Texas Red-dextran 70kD (TR-d70, 0.15% in saline, Invitrogen) was injected intravenously immediately before imaging. Then, the fresh arterial blood labeled with FITC-d2000 or albumin-FITC from the donor was injected into the cisterna magna. Imaging was obtained using a Leica SP5 2-photon imaging system (Leica Microsystems) equipped with Ti:Sapphire laser (Coherent Chameleon Ultra II, CA) and ×25/0.95 NA water-immersion objective and controlled with Leica LAS X software. After intracisternal injection, blood movement into the brain parenchyma was evaluated by 512×512 pixel image acquisition with dual-channel (FITC and Texas Red). Dynamic imaging was captured with 342ms intervals using an XYT order for 1 h. Stacks of images were acquired using a step size of 1.0μm (single stacks) to a depth of 250μm in a XYZ order (512×512 pixs). To evaluate the permeability of FITC-d2000 or albumin-FITC in PVS after SAH, imaging planes at 100 to 120μm below the cortical surface were acquired with 5min intervals between 3-dimensional stacks using an XYZT order (512×512 pixs) for 1h. To evaluate the spasm of pial arterioles and cortical microvasculature, 3 random stacks of images were acquired using a step size of 1.0μm (single stacks) to a depth of 250μm with a XYZ order (512×512 pixs) in the region of MCA.

All the images were acquired by using two-channel NDD detection with emission filter 525/50nm and 585/40nm on TCS SP5 MP System (Leica Microsystems, Mannheim, Germany). Three dimensional (3D) reconstructed images of the vascular network and quantification of vessel diameters and fluorescent average intensity were performed with an automated image-processing algorithm using LAS X software (Leica). The pial arterioles were identified according to the flow direction of RBC in *in vivo* imaging and their degree of branching was traced from the MCA (A1 to A6) (2). Individual vessel constrictions were analyzed as described previously (3).

**Administration of tissue-type plasminogen activator (tPA)**

To evaluate the potential effects of tPA on the glymphatic system after SAH, tPA (1µg in 1µl saline, totally 5µl) or saline was injected slowly into the right lateral ventricle (coordinate: AP +0mm; LR +1.75mm; DV = -3mm) at 30min after SAH. At 24 h after SAH, 10μl TR-d70 were infused into the subarachnoid CSF via cisterna magna puncture, at a rate of 2μl/min for a period of 5 minutes through a 30 gauge syringe pump. To visualize the vasculature, 0.25ml FITC-d2000 was injected intravenously immediately before imaging. The mice were imaged under the Leica SP5 two-photon microscope with ×25/0.95 NA water-immersion objectives and controlled with Leica LAS X software.

**Administration of** **Fasudil**

To evaluate the effect of vasodilator drugs, we administrated Fasudil (a rho-kinase inhibitor which decreases the myosin contraction after SAH) (4, 5) at 30min after SAH (10 mg/kg, i.p., twice per day for up to 7 days) and the control group was treated with an equal volume of saline.

**Neurological evaluation following SAH**

On day 7 after SAH a Garcia neurological scoring system (scoring scale 0 to 21), which assessed the activity, limb symmetry, climbing, balance, roprioception, vibrissae, and tactile, was used to evaluate the animal neurological behavior and function in a blinded fashion. The results were represented as the higher the neurological score, the better the outcome. The behavioral tests were performed blinded to the genotypes and the interventions. Mice were sacrificed afterwards and brain tissues were collected for further histologic evaluation.

**Immunofluorescence**

For histologic evaluation, mice were perfused transcardially with 50ml of ice-cold saline followed by 200 ml of 4% (w/v) formaldehyde in PBS. Brains were then incubated overnight in 20 to 30% sucrose and embedded in Tissue-Tek Optimal Cutting Temperature compound (Sakura Fine Technical, Tokyo, Japan) at 20°C. Brain tissue blocks were consecutively cryosectioned into 10 µm thick cross sections with a cryostat microtome (Leica Microsystems Inc., Jena, Germany).

For immunofluorescence, the frozen sections were treated with 0.3% triton and 10% anti-donkey serum for 1h at room temperature. Subsequently, the sections were incubated overnight at 4°C in dark with primary antibodies including mouse anti-NeuN (1:400, Millipore), rabbit anti-Iba-1 (1:500, Woko), goat anti-Iba-1 (1:400, abCOM), rabbit anti-GFAP (1:500, Millipore), mouse anti-α-SMA (1:100, Boster), rabbit anti-Ferritin (1:200, Matlab), mouse anti-TLR4 (1:100, Santa Cruz Biotechnology), rabbit anti-TNF-α (1:50, Boster), rabbit anti-IL-1β (1:50, Boster), and rabbit anti-MCP-1 (1:50, Boster), and then incubated with species-specific fluorescence-conjugated secondary antibodies at 37°C for 1 h in the dark. All the sections were mounted with DAPI as a nuclear stain. A Leica TCS SP5 Spectral confocal microscope was used for all immunofluorescence studies. The analysis of immunoreactivity was performed using ImageJ 2.1.4.7 software by the researcher who was blind to the experiment.

**CLARITY**

The mice brain after perfused by formalde hydeacrylamide hydrogel was extracted for processing CLARITY (6, 7). First, the brain was incubated in hydrogel monomer solution at 4°C for 6 h and then in hydrogel monomer solution without 4% PFA at 4 °C for 3 days. The brain was then embedded in polymerized hydrogel at 37°C for 3 h and cut into 2 mm-thick coronal sections with mouse brain matrix. Clarification was completed by incubation in a solution of 8% (wt/vol) SDS (Sigma) in 0.1M PBS (pH 7.5) at 37°C for 2-3 weeks, followed by washing twice for 1 day in 0.1M PBS + 0.1% Triton X-100 (PBST; Sigma).

The clarified tissue was blocked at room temperature overnight using 10% Donkey serum, followed by incubation with primary antibody including mouse anti-α-SMA (1:50, Boster), rabbit anti-Iba-1 (1:100, Woko), rabbit anti-GFAP (1:100, Millipore), or rabbit anti-fibrinogen (FIB, 1:100, Abcom ) in PBST at 37°C for 2 days. Then it was washed twice in PBST at 37°C for 1 day, and incubated in Alexa Fluor® 488 donkey anti-mouse IgG (1:200, Invitrogen) and Alexa Fluor® 555 donkey anti-rabbit IgG (1:200, Invitrogen) in PBST at 37°C for 2 days, and finally washed twice in PBST at 37°C for 1 day.

For imaging, the stained samples were placed in FocusClear (CelExplorer) at least 2h prior to imaging and were mounted on cover glass-bottomed dishes (Willco) and imaged using a water immersion objective with a magnification of 25× and N.A. of 0.95 (Leica SP5). Image stacks were acquired with a z-step spacing of 0.5μm or 1μm controlled with Leica LAS X software.

**Western-blot analysis**

For protein analyses, mice were perfused transcardially with 50ml ice-cold saline on day 7 after SAH. Brains were then rapidly removed and placed in a brain blocker on ice and further processed for protein extraction with Total Protein Extraction Kit (Beyotime Institute of Biotechnology, China) following the recommendations of the manufacturer. Total protein samples were separated on 10% SDS/PAGE gels and electrotransferred to PVDF membranes at 16V for 30min. The membranes were blocked with 5% fat-free milk in TBS containing 0.05% Tween 20 (TBST, 2h at room temperature) and washed in TBST. The blots were probed with primary antibodies including rabbit anti-GFAP (1:1000, Bioworld), mouse anti-TLR4 (1:1000, Santa Cruz Biotechnology), rabbit anti-TNF-α (1:1000, Proteintech), and rabbit anti-AQP4 (1:250, Alomone) at 4°C overnight, then washed for 30min in TBST and incubated with horseradish peroxidase-conjugated goat anti-mouse IgG or goat anti-rabbit IgG (both at 1:1000, Forevergen) for 2h at room temperature, and visualized using chemiluminescence (ECL, Forevergen). OD was analyzed using Image J Software, and results normalized to β-actin were expressed as fold change relative to the control. Each experiment was performed at least three times with representative gels shown.

**Clinical data**

We retrospectively analyzed 24 cases of hospitalized aneurysmal SAH patients who simultaneously underwent the examination of unenhanced cerebral computed tomography scan (CT scan), cerebral CT angiography (CTA) and contrast-enhanced cerebral CT scan on admission from April 2013 to April 2015 in the First affiliated Hospital of Sun Yat-Sen University. The diagnosis of SAH was based on the patient's medical history and clinical manifestation and verified by an examination of unenhanced cerebral CT scan. Among these 24 cases, there were 13 males and 11 females, with ages ranging from 19 to 68 years old. All CT examinations were performed using a 320-detector row volume CT system (Aquilion ONE; Toshiba Medical Systems, Nasu, Japan), with a detector width of 160 mm. Unenhanced CT was initially undertaken to obtain the mask image for subtraction. Contrast enhancement was provided by the intravenous antecubital administration of a 50 ml bolus of non-ionic iodinated contrast material (iopromide, 370 mg iodine/ml, Ultravist, Bayer Schering, Berlin, Germany) at a flow rate of 6 ml/s, followed by 30 ml saline solution. CT parameters were as follows: 0.75 s/r gantry rotation speed; 320×0.5 mm detector width; 0.25 mm reconstruction interval; 512×512 matrix; 180-240 mm field of view; 80kV tube voltage; 350mA (non-enhanced image); 150mA (contrast-enhanced image) tube current. The CTA was initiated 7s after the start of an intravenous infusion. The CTA volume data were obtained by subtracting the mask image volume data from the non-subtracted CTA volume data. CTA volume data were transported to a professional workstation (VITREA).

For human brain tissue sample data, the brain sample of SAH was isolated from a SAH patient (A 27-year-old female presented with a sudden severe headache was admitted to hospital on April 6, 2015. CT scan revealed a diffuse subarachnoid hemorrhage. CTA showed an aneurysm of the left former traffic artery) who received intracranial aneurysm clipping with right pterional approach on day 1 after SAH at Tangdu Hospital, Xi’An. The brain sample of control was isolated from a drug-resistant mesial epilepsy patient (A 21-year-old female presented with repeated psychomotor seizure for 3 years was admitted to hospital on march 24, 2015) who received temporal lobectomy on March 27, 2015 at Tangdu Hospital, Xi’An. This part of the study complied with the guidelines of the Declaration of Helsinki and was approved by the Human Ethics Committee of Tangdu Hospital, Xi’An, China. The written informed consent was obtained from the subject. The brain tissue sample was then proceeded for hematoxylin-eosin (HE) staining.

**Statistical Analysis**

All the data were presented as the mean ± SEM. Statistical analysis was performed with SPSS 17.0 (SPSS, Inc., Chicago, IL, USA). Differences in perivascular Iba-1 and GFAP immunofluorescence between 2 groups were compared by an unpaired *t* test. Differences in neurological scoring and western-blot analysis were evaluated by one-way ANOVA followed with Tukey’s post hoc test for multiple comparisons. P <0.05 was considered to be statistically significant.

**References:**

1. Koide M, Bonev AD, Nelson MT, Wellman GC. Inversion of neurovascular coupling by subarachnoid blood depends on large-conductance Ca2+-activated K+ (BK) channels. *Proc Natl Acad Sci U S A* 2012 2012-05-22; **109** (21)**:** E1387-E1395.

2. Strahler AN. GEOMORPHIC SIGNIFICANCE OF VALLEYS AND PARKS OF THE KAIBAB AND COCONINO PLATEAUS, ARIZONA. *SCIENCE* 1944 1944-09-08; **100** (2593)**:** 219-220.

3. Friedrich B, Muller F, Feiler S, Scholler K, Plesnila N. Experimental subarachnoid hemorrhage causes early and long-lasting microarterial constriction and microthrombosis: an in-vivo microscopy study. *J Cereb Blood Flow Metab* 2012 2012-03-01; **32** (3)**:** 447-455.

4. Satoh S, Takayasu M, Kawasaki K, Ikegaki I, Hitomi A, Yano K*, et al.*. Antivasospastic effects of hydroxyfasudil, a Rho-kinase inhibitor, after subarachnoid hemorrhage. *J PHARMACOL SCI* 2012 2012-01-20; **118** (1)**:** 92-98.

5. Zhao J, Zhou D, Guo J, Ren Z, Zhou L, Wang S*, et al.*. Efficacy and safety of fasudil in patients with subarachnoid hemorrhage: final results of a randomized trial of fasudil versus nimodipine. *Neurol Med Chir (Tokyo)* 2011 2011-01-20; **51** (10)**:** 679-683.

6. Chung K, Wallace J, Kim SY, Kalyanasundaram S, Andalman AS, Davidson TJ*, et al.*. Structural and molecular interrogation of intact biological systems. *NATURE* 2013 2013-05-16; **497** (7449)**:** 332-337.

7. Tomer R, Ye L, Hsueh B, Deisseroth K. Advanced CLARITY for rapid and high-resolution imaging of intact tissues. *NAT PROTOC* 2014 2014-07-01; **9** (7)**:** 1682-1697.
