# Supplementary material for: Paravascular pathways contribute to vasculitis and neuroinflammation after subarachnoid hemorrhage independently of glymphatic control
Source: Cell Death Dis. 2016 Mar 31;7(3):e2160–. doi: 10.1038/cddis.2016.63 (PMC4823962; doi:10.1038/cddis.2016.63)
Supplement: Supplementary Information 2 [file cddis201663x2.doc]

**Abbreviations**

SAH Subarachnoid hemorrhage

tPA tissue-type plasminogen activator

DCI delayed cerebral ischemia

CVS cerebral vasospasm

CNS central nervous system

GS glymphatic system

PVS paravascular space (or perivascular space in somewhere)

AQP4 aquaporin-4

ISF interstitial fluid

TBI traumatic brain injuries

CBF cerebral blood flow

DC decompressive

CSF cerebrospinal fluid

aCSF artificial cerebrospinal fluid

MCA middle cerebral artery

HO-1 Heme oxygenase-1

TLR4 Toll-like receptor 4

NF-κB nuclear factor-κB

CT computed tomography

CTA CT angiography
