# Supplementary figures and images for: Paravascular pathways contribute to vasculitis and neuroinflammation after subarachnoid hemorrhage independently of glymphatic control
Source: Cell Death Dis. 2016 Mar 31;7(3):e2160–. doi: 10.1038/cddis.2016.63 (PMC4823962; doi:10.1038/cddis.2016.63)

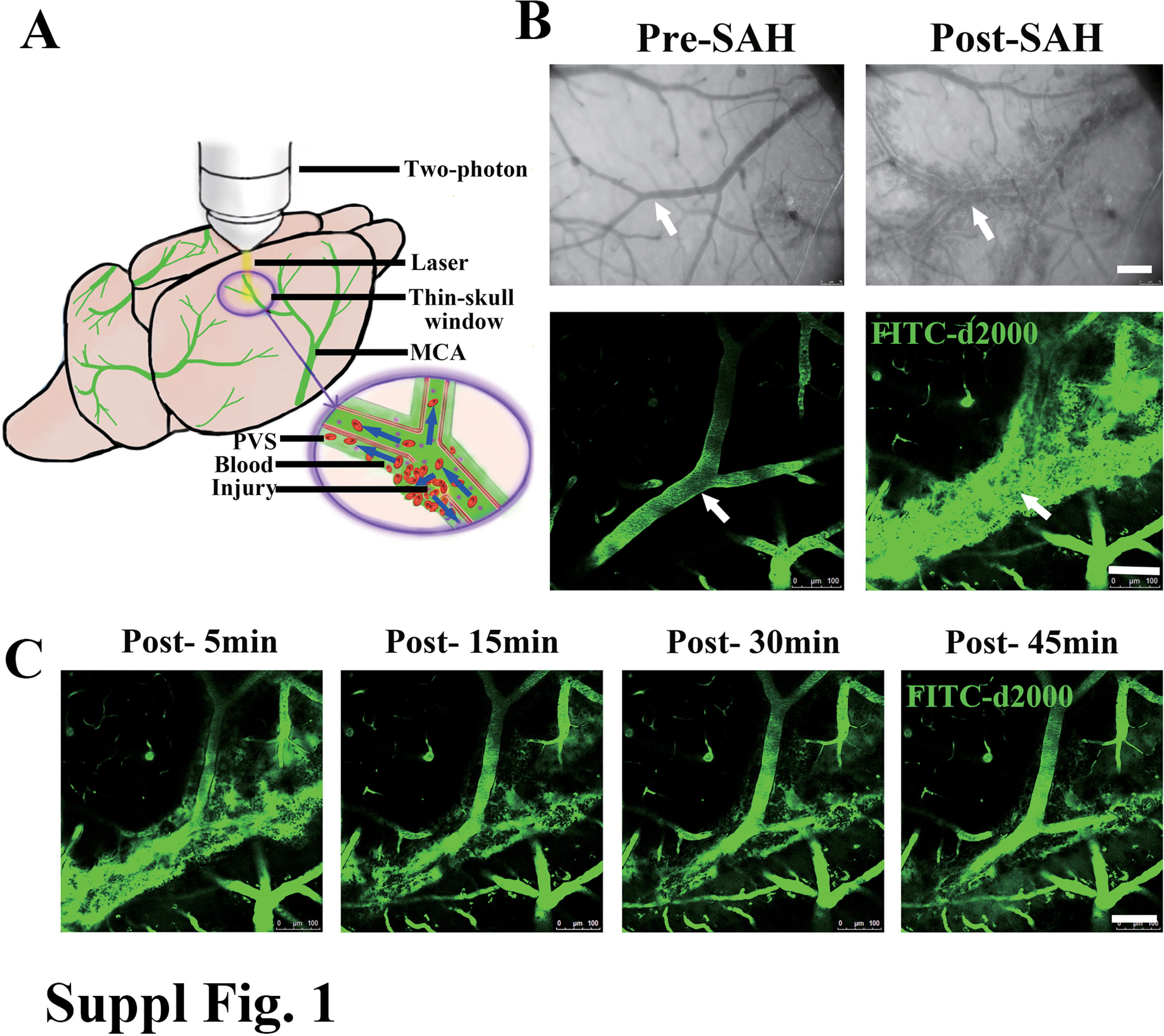

Supplement: Supplementary Figure 1 [file cddis201663x3.tif]

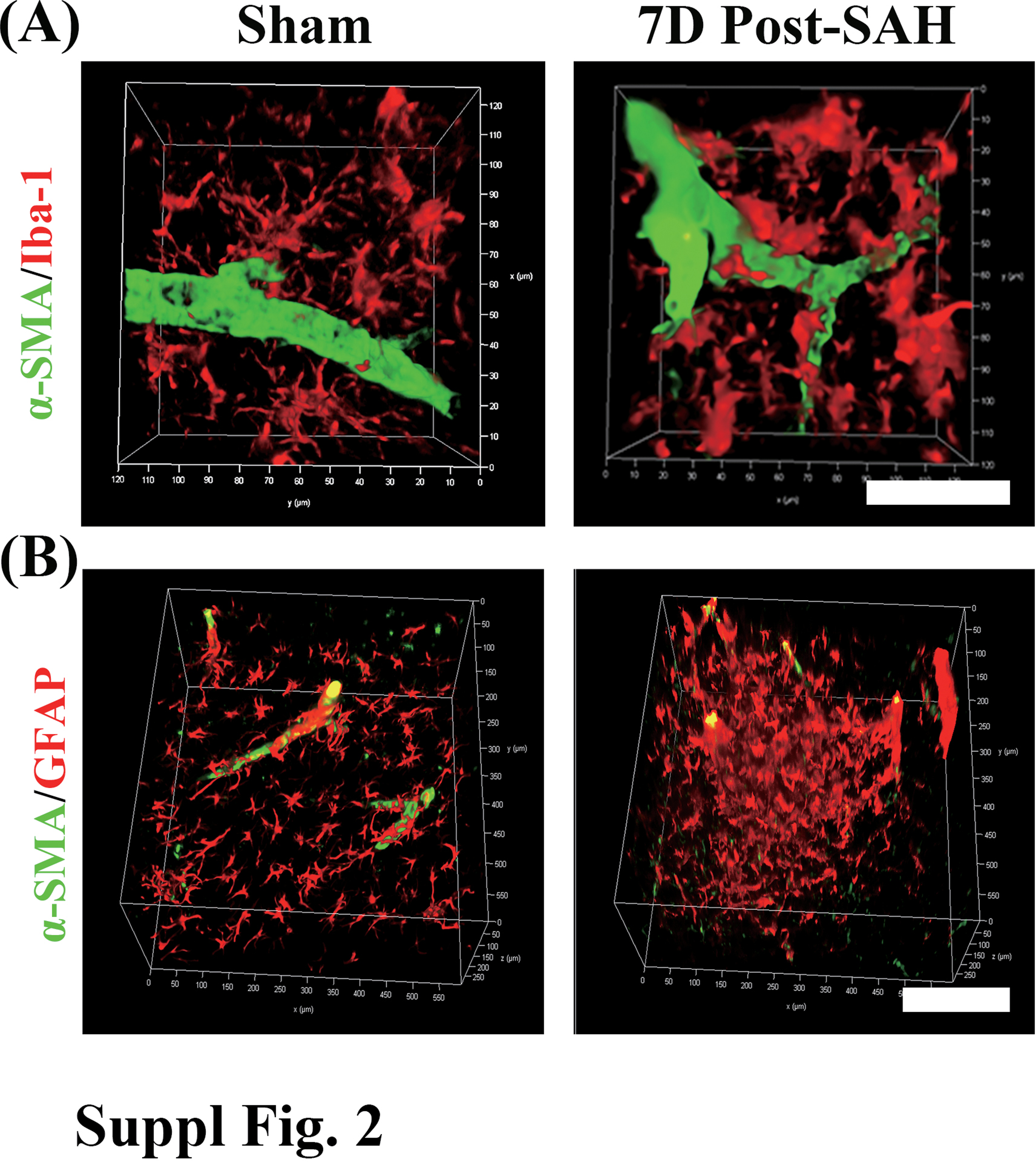

Supplement: Supplementary Figure 2 [file cddis201663x4.tif]

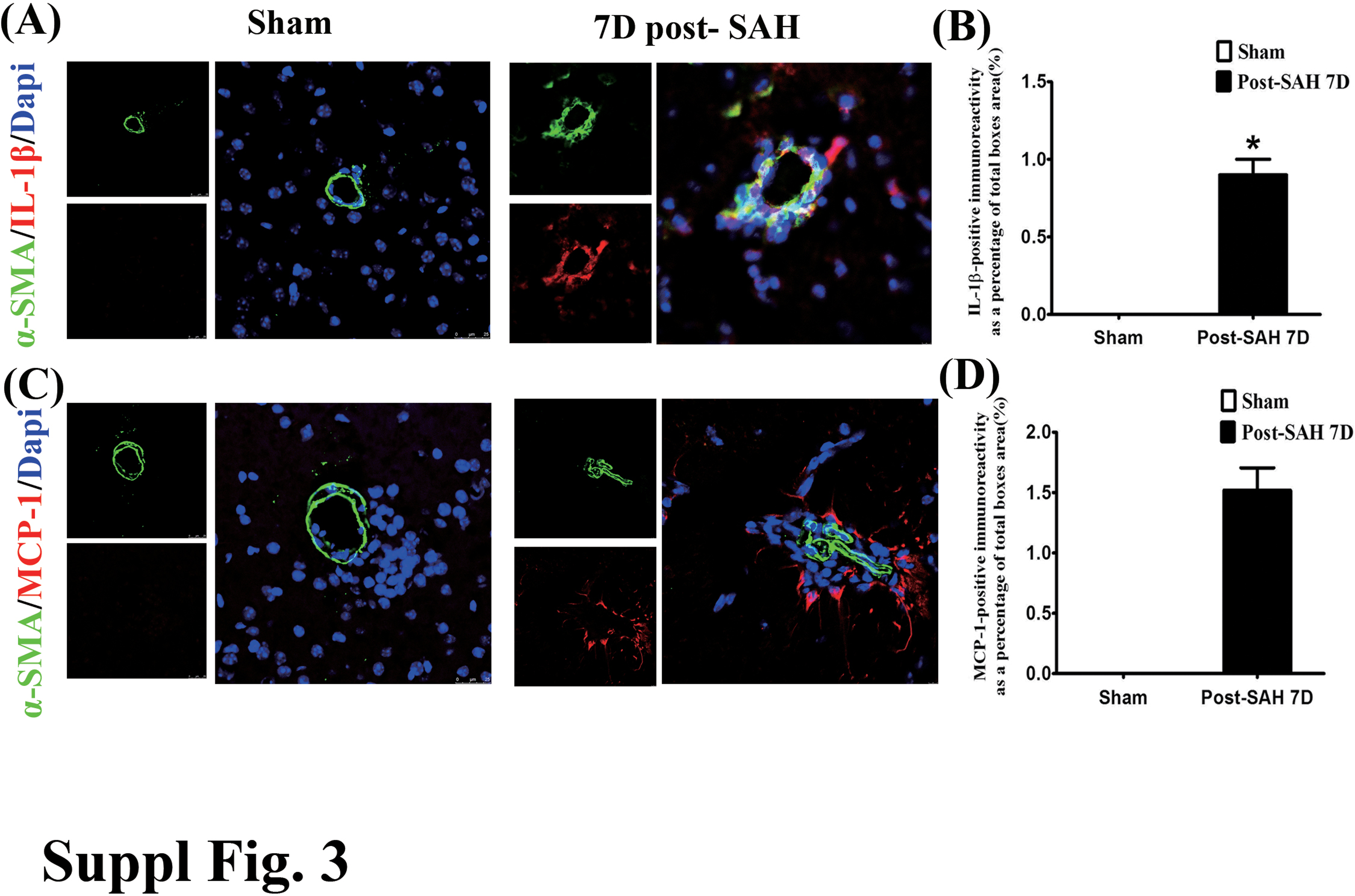

Supplement: Supplementary Figure 3 [file cddis201663x5.tif]

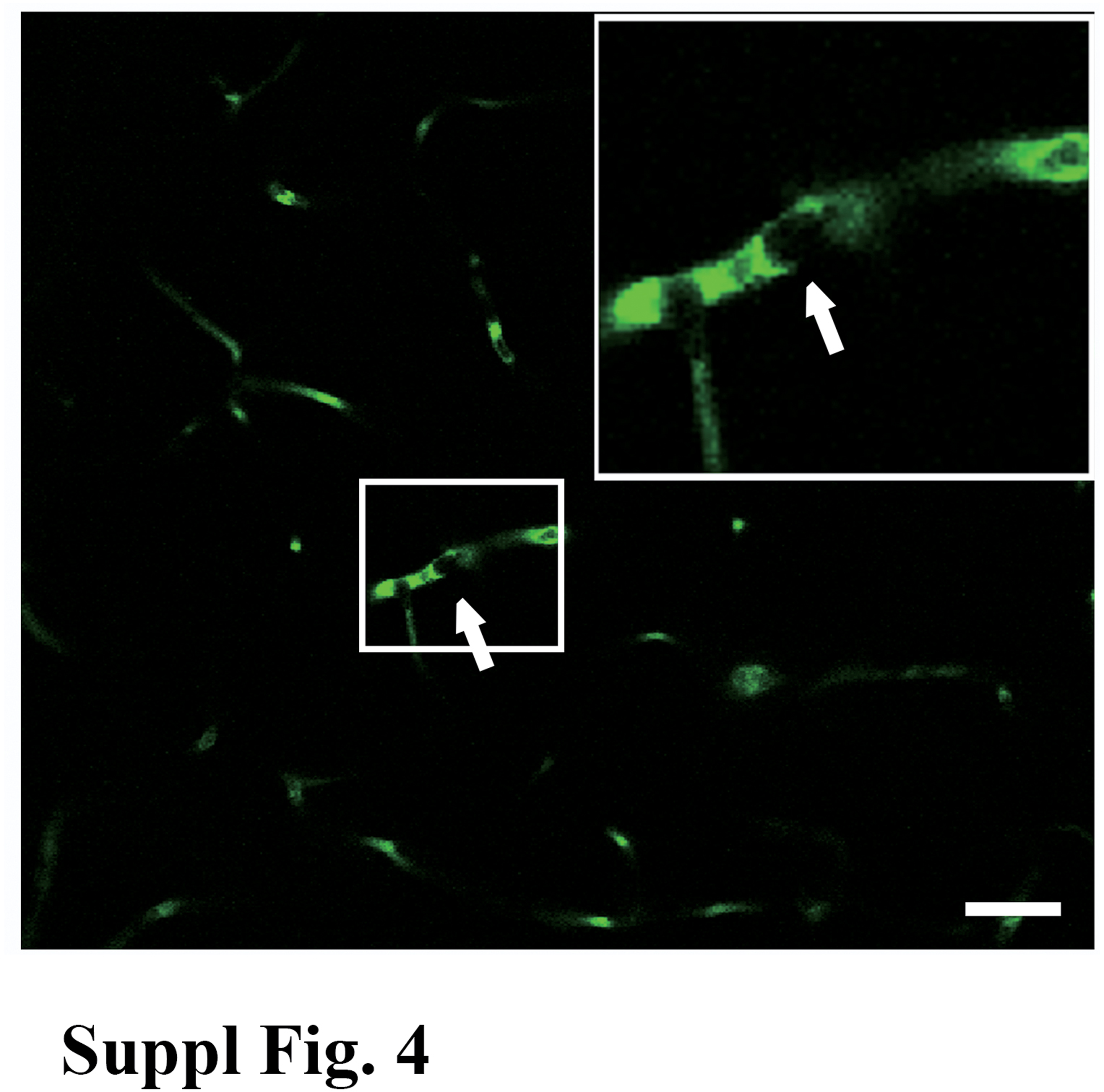

Supplement: Supplementary Figure 4 [file cddis201663x6.tif]
