# Supplementary material for: Paravascular pathways contribute to vasculitis and neuroinflammation after subarachnoid hemorrhage independently of glymphatic control
Source: Cell Death Dis. 2016 Mar 31;7(3):e2160–. doi: 10.1038/cddis.2016.63 (PMC4823962; doi:10.1038/cddis.2016.63)
Supplement: Supplementary Figure and Video Legends [file cddis201663x10.doc]

Suppl Fig 1. [Blood flows into the GS after SAH](http://mts-ncomms.nature.com/ncomms_files/2015/07/26/User/AppData/Roaming/Foxmail7/AppData/Local/Microsoft/Windows/Temporary Internet Files/Content.IE5/AppData/Local/Microsoft/Windows/Temporary Internet Files/Content.IE5/AppData/Local/Microsoft/Windows/Temporary Internet Files/Content.IE5/9ED35ZPN/blood flow along the PVS.avi) induced by femtosecond laser ablation on the pial artery in mice. (A) Schematic illustrating a SAH model established by using focused femtosecond laser pulses to induce pial artery rupture in mice. (B) Representative images showing the appearance of the paravascular pathway surrounding the pial artery after SAH by camera and the distribution of blood which was labeled by intravenous injection of FITC-d2000 in the PVS after SAH induced by femtosecond laser ablation on the pial artery with 2 photon imaging. (C) A temporal recording on the dynamical distribution of FITC-d2000-labeld blood in the PVS after SAH. Scale bar: 100μm.

Suppl Fig 2. Microglial cells and astrocytes were pronouncedly activated in the perivascular parenchyma on day 7 after SAH in mice. (A) Representative CLARITY images showing the activation of microglial cells in the perivascular parenchyma on day 7 after SAH in mice. (B) Representative CLARITY images showing the activation of astrocytes in the perivascular parenchyma on day 7 after SAH in mice. Scale bar: 50μm for A and 200μm for B.

Suppl Fig 3. The expression of inflammatory factors in the perivascular parenchyma on day 7 after SAH in mice. (A and B) Representative of mages showing a higher expression of IL-1β in the perivascular parenchyma on day 7 after SAH in mice (N=5, P<0.05). (C and D) Representative of mages showing a higher expression of MCP-1 in the perivascular parenchyma on day 7 after SAH in mice (N=5, P<0.05). Scale bar: 45μm.

Suppl Fig 4. Representative images showing the formation of microemboli (arrows) in the cerebral capillary network at 6 h after SAH in mice (The bloodstream is defined by intravenously injected FITC-d2000). Scale bar: 100μm.

Video 1. A real-time recording shows [subarachnoid blood flows into the PVS after SAH](http://mts-ncomms.nature.com/ncomms_files/2015/07/26/User/AppData/Roaming/Foxmail7/AppData/Local/Microsoft/Windows/Temporary Internet Files/Content.IE5/AppData/Local/Microsoft/Windows/Temporary Internet Files/Content.IE5/AppData/Local/Microsoft/Windows/Temporary Internet Files/Content.IE5/9ED35ZPN/blood flow along the PVS.avi) in mice using a 2 photon imaging system.

Video 2. A real-time recording shows the formation of microemboli in the cerebral capillary network at 6 h after SAH in mice using a 2 photon imaging system.

Video 3. A real-time recording shows the vasospasm induced by blood clotting in the GS after SAH in mice using a 2 photon imaging system.
